# The Crystal Structure of the YknZ Extracellular Domain of ABC Transporter YknWXYZ from *Bacillus amyloliquefaciens*

**DOI:** 10.1371/journal.pone.0155846

**Published:** 2016-05-31

**Authors:** Yongbin Xu, Jianyun Guo, Lulu Wang, Rui Jiang, Xiaoling Jin, Jing Liu, Shengdi Fan, Chun-Shan Quan, Nam-Chul Ha

**Affiliations:** 1 Department of Bioengineering, College of Life Science, Dalian Nationalities University, Dalian 116600, Liaoning, China; 2 Laboratory of Biomedical Material Engineering, Dalian Institute of Chemical Physics, Chinese Academy of Sciences, Dalian 116023, Liaoning, China; 3 School of Biological Engineering, Dalian Polytechnic University, Dalian 116034, Liaoning, China; 4 College of Life and Health Sciences, Northeastern University, Shenyang 110004, Liaoning, China; 5 Department of Agricultural Biotechnology, College of Agriculture and Life Sciences, Seoul National University, Gwanak-gu, Seoul 151–742, Republic of Korea; University of Queensland, AUSTRALIA

## Abstract

*Bacillus* possesses the peptide toxin Sporulation-Delaying Protein (SDP), which can kill cells within a biofilm to support continued growth, thereby delaying the onset of biofilm sporulation. The four-component transporter YknWXYZ acts as a major SDP efflux pump to protect cells against the endogenous SDP toxin, for which YknYZ is a non-canonical ATP-binding cassette (ABC)-type transporter. YknYZ consists of the following two components: (1) an individual protein (YknY) and (2) a respective permease (YknZ). To date, the crystal structure, molecular function, and mechanism of action of the integral membrane protein YknZ remain to be elucidated. In this study, to characterize the structural and biochemical roles of YknZ in the functional assembly of YknWXYZ, we predicted and overexpressed the YknZ extracellular domain. We determined the crystal structure of *B*. *amyloliquefaciens* YknZ at a resolution of 2.0 Å. The structure revealed that the YknZ extracellular region exhibits significant structural similarity with the MacB periplasmic domain, which is a non-canonical ABC-type transporter in the tripartite macrolide-specific efflux pump in Gram-negative bacteria. We also found that the YknZ extracellular domain can directly bind to an extracellular component of YknX. This structural and biochemical study provides insights into the assembly of YknWXYZ, which may be relevant to understanding cannibalistic peptide toxin resistance in *Bacillus* and controlling bacterial growth.

## Introduction

The Gram-positive bacterium *Bacillus subtilis* cannibalistic toxin Sporulation-Delaying Protein (SDP) kills a subset of *Bacillus* cells within a biofilm to support continued growth, thereby delaying the onset of biofilm sporulation [1,2]. Mature SDP is a product of the *sdpC* gene, and recent analyses of the structure and activity of SDP have shown that this toxin is a 42-amino-acid peptide containing a single intramolecular disulfide bond [3]. The four-component transporter YknWXYZ in *Bacillus* has been implicated in the protection against SDP-dependent killing by expelling invading SDP toxin from the cytosol [4].

YknWXYZ contains the ATP-binding cassette (ABC) transporter YknYZ, in which an individual protein YknY dimer associates with a respective permease (YknZ) that utilizes ATP hydrolysis as a driving force [5]. The activity of YknYZ strictly depends on two accessory proteins, YknX and YknW. The membrane fusion protein (MFP) YknX is located in the extracellular region and anchors to the cytoplasmic membrane via an N-terminal anchor [6]. YknX is anchored to the cytoplasmic membrane via an uncleavable N-terminal signal peptide and is connected to YknZ [4]. YknW, a Yip1 family protein that is highly conserved in *Bacillus* species, can modulate the assembly of the YknXYZ complex by forming a composite interface with YknX and YknYZ [4].

Recently, YknWXYZ homologues were identified in various Gram-negative bacteria [4]. In *Escherichia coli*, macrolide-specific efflux transporters, such as MacAB-TolC, have also been implicated in enterotoxin secretion and macrolide antimicrobial compound discharge [7,8]. The MFP MacA connects an ABC-type transporter, MacB, and the outer membrane factor TolC to pump out macrolide antibiotics [9]. The non-canonical ABC-type transporter MacB was first identified as a macrolide antibiotic-specific transporter in *E*. *coli* [10]. Notably, full-length MacB is believed to act as a homodimeric protein and has a novel architecture that consists of an N-terminal nucleotide binding domain, a four-transmembrane segment, and two putative extracellular regions [7,8,11]. Some research groups have revealed that the inner membrane component of MacB directly interacts with MFP MacA [12]. In Gram-negative bacteria, MacA exists as a funnel-like hexamer and forms an intermeshing cogwheel by interacting with TolC [9,13,14]. Several lines of evidence for a mechanism for the ABC-type transporter MacB have been suggested [12], but the reported studies have focused on Gram-negative bacteria instead of Gram-positive bacteria.

To gain insight into the functional and structural properties of the YknZ respective permease, we aimed to determine the three-dimensional structure of YknZ. Here, we present the first crystal structure of the extracellular domain of YknZ from *Bacillus amyloliquefaciens*. Biochemical experiments demonstrated that the extracellular domain of YknZ has a strong affinity for the MFP YknX.

## Materials and Methods

### Construction of YknZ ED and YknX expression plasmids

To construct the plasmid expressing YknZ ED, DNA fragments encoding *B*. *amyloliquefaciens* YknZ (residues 44–273, 24.8 kDa) were amplified by PCR from *B*. *amyloliquefaciens* genomic DNA with the following oligonucleotides: (1) 5'-ggg**ccatgg**atggcggagagcaaatgctg-3' and (2) 5'-ggg**ctcgag**ttacgtcgtcataacggtcgt-3' (restriction sites are in bold). The insert was cloned into the pPROEX-HTA *E*. *coli* expression vector (Invitrogen, USA) using the restriction enzymes *Nco*I and *Xho*I. The plasmids contained a hexa-histidine tag-containing region (Met-Ser-Tyr-Tyr-His-His-His-His-His-His-Asp-Tyr-Asp-Ile-Pro-Thr-Thr) and a tobacco etch virus (TEV) protease cleavage site (Glu-Asn-Leu-Tyr-Phe-Gln) at the N-terminus of the YknZ ED. To construct the plasmid expressing YknX for the *in vitro* binding assay, DNA fragments encoding *B*. *amyloliquefaciens* YknX (residues 34–378) were similarly cloned using the following primers: (1) 5'-ggg**ccatgg**atgggaaaaaaatcgaaacc-3' and (2) 5'-ggg**ctcgag**ctatgctttcacatccgttcc-3' (restriction sites are in bold).

### Expression and purification of the recombinant YknZ ED

Expression and purification of the recombinant proteins of YknZ ED and YknX were performed using similar protocols. The constructs were transformed into BL21 cells (DE3), and the proteins were expressed in the cytosol as hexa-histidine-tag fusion proteins. A single colony was used to inoculate 25 ml of LB broth supplemented with 50 μg ml^-1^ ampicillin, which was incubated overnight at 310 K with shaking. Cultured YknZ ED expression cells were then transferred to 1.5 liters of LB broth with 50 μg ml^-1^ ampicillin at 310 K until reaching an OD_600_ between 0.6 and 0.8. In particular, the recombinant *Ba* YknZ ED protein was expressed using M9 medium supplemented with an amino acid mixture containing L-(+)-selenomethionine at 303 K until reaching an OD_600_ between 0.6 and 0.8. After induction with 0.5 mM isopropyl-d-thiogalactoside, the cultures were grown for 6 h at 303 K. The BL21 cells (DE3) were harvested by centrifugation and then stored at 193 K until use. Cell pellets were resuspended in a lysis buffer containing 20 mM Tris (pH 8.0), 150 mM NaCl, and 2 mM 2-mercaptoethanol, and the cells were lysed by sonication on ice. The cell debris was removed by centrifugation at 45,000 g for 30 min at 277 K. The resulting supernatant was then stirred for 30 min at 4°C and loaded onto a Ni-NTA affinity resin (Qiagen, Netherlands). After the resin was washed with lysis buffer supplemented with 20 mM imidazole, the recombinant hexa-histidine-tagged *Ba* YknZ ED was eluted with 30 ml of lysis buffer supplemented with 250 mM imidazole. After heating, the eluted fractions were determined by SDS-PAGE analysis and pooled, and 2-mercaptoethanol was added to a final concentration of 10 mM. The hexa-histidine tag was removed by incubating the recombinant protein overnight at 298 K with recombinant TEV protease. The solution was diluted 3-fold with 20 mM Tris (pH 8.0) buffer and then loaded onto a Q anion-exchange column (HiTrap-Q; GE Healthcare, USA). The protein was eluted from the column using a linear gradient of 0 to 1 M NaCl in 20 mM Tris buffer (pH 8.0). The collected fractions containing the target recombinant proteins were further purified and separated on a HiLoad Superdex 200 gel-filtration column (GE Healthcare, USA) pre-equilibrated with a buffer consisting of 20 mM Tris-HCl (pH 8.0), 150 mM NaCl, and 2 mM 2-mercaptoethanol. The fractions corresponding to the peaks were subjected to SDS-PAGE on a 15% acrylamide gel and stained with Coomassie blue. The purified proteins were concentrated to 12 mg ml^-1^ and stored at 193 K until use.

### Circular dichroism (CD) spectroscopy

To evaluate the structural integrity of the *Ba* YknZ ED recombinant protein, CD spectroscopy was performed using a JASCO-J1500 spectropolarimeter (JASCO, Japan). Concentrated *Ba* YknZ ED protein and BSA were diluted to 0.1 mg/ml in 20 mM HEPES (pH 7.5) buffer containing 150 mM NaCl. The absorption spectra were measured in the spectral range between 200 and 280 nm at 298 K. Three consecutive scans were performed and averaged, and the solvent signal was subtracted from all of the spectra.

### Crystallization, data collection, and refinement structure

The pooled, purified *Ba* YknZ ED-SeMet samples were concentrated to 12 mg ml^-1^ using a Vivaspin centrifugal concentrator fitted with a 10 kDa molecular-weight cutoff filter (Millipore, USA) prior to crystallization. The search for crystallization conditions was performed with a Crystal Screen HT high-throughput sparse-matrix screening kit (Hampton Research, USA) using the sitting-drop vapor-diffusion method at various temperatures (277, 279, 288 and 295 K). The final crystallization conditions consisted of 22.5% (*w*/*v*) polyethylene glycol 4000, 0.125 M ammonium acetate, and 0.1 M sodium acetate trihydrate (pH 4.6) at 288 K. Immediately after the crystal was removed from the drop, it was soaked in 10 μl of cryoprotectant solution consisting of well solution supplemented with 25% (*v*/*v*) glycerol for 3 to 5 s and was then flash-cooled in liquid nitrogen.

We used a single-wavelength anomalous dispersion (SAD) phasing method to resolve the structure. Diffraction data were measured to 2.0 Å, with the wavelength tuned to 0.9795 Å, using an ADSC Q310 CCD detector on the BL17U beamline at the Shanghai Synchrotron Radiation Facility (SSRF) (Shanghai, People’s Republic of China). All of the data were collected at 100 K using a nitrogen stream. An oscillation range of 1° per frame over a 360° rotation was implemented, the exposure time was 5 sec per frame, and the detector distance was 340 mm. The intensity data were processed, merged and scaled using the *HKL*-2000 program [15].

The structure was solved via SAD-phasing using AUTOSOL from the PHENIX package, and the nearly complete model was improved after refinement with REFINE from the PHENIX package [16]. The *Ba* YknZ ED model was refined to convergence, resulting in an R factor of 18.1% and an R_free_ of 24.9% to a resolution of 2.0 Å. Ramachandran plots and root-mean-square deviations from ideality for bound angles were determined using PHENIX [16]. One molecule of *Ba* YknZ ED-SeMet constituted the asymmetric unit, and each contained six SeMet residues. The model building and fit to electron-density maps were performed with the graphics software COOT [17]. All of the figures containing molecular structures were prepared using PyMOL [18]. The data collection and refinement statistics are given in Table 1.

**Table 1:** Crystallography data and refinement statistics.

**Data collection**	
**Space group**	*P*2_1_2_1_2_1_
**Cell dimensions**	
***a*, *b*, *c* (Å)**	44.17, 46.15, 87.54
***α*, *β*, *γ* (°)**	90.00, 90.00, 90.00
**Resolution range (Å)**	41.0–2.0 (2.08~2.04)
**R**_**merge**_ **(%)**	11.8 (80.9)
**Completeness (%)**	99.8 (99.7)
**Multiplicity**	7.0 (6.9)
**Average *I/σ(I)***	28.8 (2.7)
**Model refinement**	
**R**_**factor**_**/ R**_**free**_ **(%)**	19.44/24.73
**No. of atoms**	
**Protein**	1437
**Water**	98
**Average B factor (Å**^**2**^**)**	35.07
**R.m.s.d. from ideal**	
**Bond lengths (Å)**	0.010
**Bond angles (◦)**	1.157
**Ramachandran plot**	
**Preferred (%)**	96.69%
**Outliers (%)**	0.00%
**PDB code**	5F9Q

R_merge_ = ∑_*hkl*_∑_*i*_| *I*_*i*_*(hkl)- < I(hkl) >*|∑_*hkl*_∑_*i*_| *I*_*i*_*(hkl)*, where I(hkl) is the intensity of reflection *hkl*, ∑_*hkl*_ is the sum over all reflections and ∑_*I*_ is the sum over *i* measurements of reflection *hkl*. R_factor_ = ∑_*hkl*_|*F*_o_-*F*_c_|/∑_*hkl*_|*F*_o_| for all data with *F*_o_ *>* 2σ(*F*_o_), excluding data used to calculate *R*_free_. R_free_ = ∑_*hkl*_|*F*_o_-*F*_c_|/∑_*hkl*_|*F*_o_| for all data with *F*_o_ *>* 2σ(*F*_o_) that were excluded from refinement.

### Size exclusion chromatography for the analysis of the *Ba* YknZ ED oligomeric state

Size-exclusion chromatography was performed to determine whether *Ba* YknZ ED forms dimeric structures in solution. Purified *Ba* YknZ ED protein (0.3 mg) was injected at a flow rate of 0.5 ml/min into a Superdex 200 10/300 GL column (GE Healthcare) that was pre-equilibrated with column buffer (20 mM Tris buffer pH 8.0, 150 mM NaCl, 2 mM 2-mercaptoethanol) at room temperature (295 K). The same column buffer was also used for the elution. The molecular weight of each protein was estimated according to the elution volume.

### In vitro binding assay for *Ba* YknZ ED and *Ba* YknX

*Ba* YknZ ED-coupled resin was prepared using CNbr-activated resin (50 μg; GE Healthcare) and *Ba* YknZ ED protein (0.5 mg) according to the manufacturer’s instructions; the *Ba* YknZ ED-uncoupled CNbr-activated region was similarly used as a control. Purified YknX protein (0.6 mg) and the coupled resin (20 μl) or uncoupled resin were incubated in 200 μl of buffer (20 mM HEPES pH 7.5, 150 mM NaCl, and 2 mM 2-mercaptoethanol) for 2 h at 277 K, and the mixture was then centrifuged at 2000 rpm for 2 min. The resin was collected and thoroughly washed five times with 500 μl of 20 mM HEPES (pH 7.5) buffer containing 150 mM NaCl and 2 mM 2-mercaptoethanol. To analyze the YknX bound to *Ba* YknZ ED protein, the beads were boiled with SDS sample buffer, applied to SDS-PAGE on a 15% gel and stained with Coomassie blue.

## Results

### Plasmid construction, expression, and purification of the recombinant *B*. *amyloliquefaciens* YknZ ED

**Fig 1:**
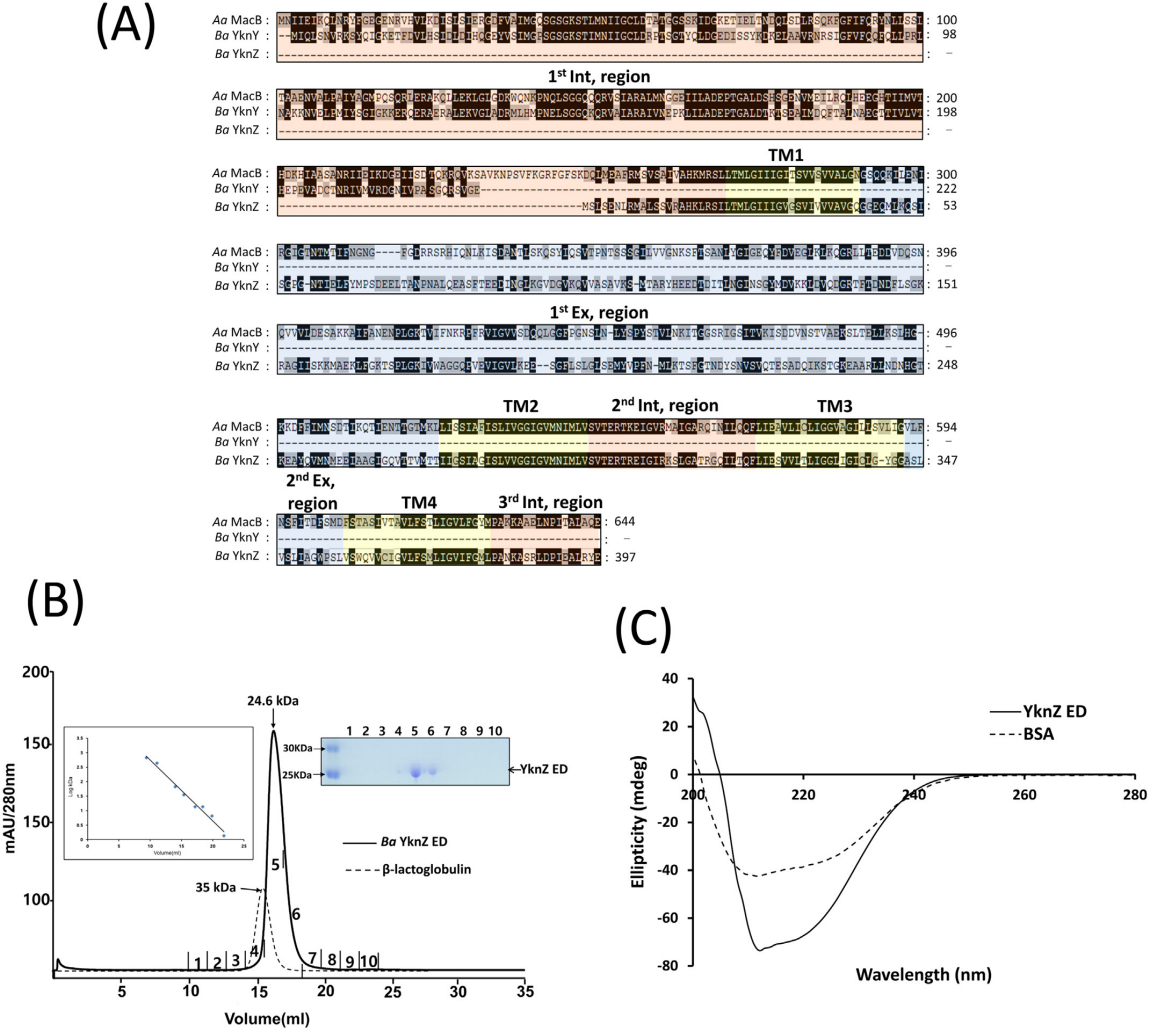
Putative YknZ ED. (A) Sequence alignment of the *Aa* MacB, *Ba* YknY, and *Ba* YknZ amino acid sequences, which were obtained from the National Center for Biotechnology Information (NCBI). The alignment was performed using CLUSTALX [20]. The intracellular regions, transmembrane segments, and periplasmic regions are highlighted in orange, yellow, and blue, respectively. The four transmembrane segments (TM1-TM4) are indicated in the sequence or above the sequence. The two extracellular domains (1^st^ Ex 2^nd^ Ex regions) are labeled above the sequences. The conserved residues are highlighted. (B) Circular dichroism spectra of *Ba* YknZ ED (YknZ ED) and bovine serum albumin (BSA). (C) *Ba* YknZ ED is monomeric in solution. Elution profiles of *Ba* YknZ ED from a size-exclusion chromatographic column (Superdex 200 10/300 GL column) shown with estimated molecular weights. Prior to the experiment, the column was calibrated with standard molecular weight markers inset, and the straight line represents a linear fit. The numbers correspond to the fractions indicated on each chromatogram. The SDS-PAGE analyses of the fractions are shown. When the standard size markers indicated by arrows were considered, the molecular weight of the *Ba* YknZ ED was calculated as ~ 24.6 kDa, which is estimated to be the monomer of *Ba* YknZ ED. The elution profile of β-lactoglobulin (35 kDa) on a size-exclusion column is shown as a dotted line.

The respective permease YknZ is a 397-amino-acid membrane protein. Functional and structural studies of the YknZ native state usually require the overexpression of full-length YknZ; however, conducting these studies is challenging because the expression yield of the full-length YknZ construct is usually too low [4]. In this study, we found that YknZ has a novel architecture that consists of a four-transmembrane segment and two putative extracellular regions (Fig 1A, residues 44–273 and residues 345–358) as predicted by analysis using TMHMM Server V. 2.0 (www.cbs.dtu.dk) [19]. The second YknZ extracellular region (residues 345–358) appeared to be too short to form a structured motif because it consists of only 14 residues. Thus, because only the first YknZ extracellular region was expected to form a structured domain, the first YknZ extracellular region was named the extracellular domain (ED) in this study. To determine whether *Ba* YknZ ED forms dimeric structures in solution, we measured the molecular size of *Ba* YknZ ED using size-exclusion chromatography. We obtained data suggesting that the *Ba* YknZ ED protein is eluted as a monomer on a size-exclusion column (Fig 1B).

The YknZ ED was constructed and successfully overexpressed, with a yield of 12 mg from one liter of Luria-Bertani (LB) medium culture. The purity of the protein sample was determined to be > 93% according to Coomassie staining following sodium dodecyl sulfate-polyacrylamide gel electrophoresis (SDS-PAGE) (S1 Fig). To ascertain whether *Ba* YknZ ED behaved as a well-folded protein, the secondary structure of *Ba* YknZ ED was characterized by circular dichroism (CD) spectroscopy. The CD spectrum was very similar to the bovine serum albumin (BSA) CD spectrum (Fig 1C). The results indicated that YknZ ED has structural integrity because its spectrum matches the expected structure of YknZ in the absence of the membrane-embedded domain.

### Structural determination of the recombinant *Ba* YknZ ED

The purified recombinant YknZ ED protein sample was successfully crystallized under several conditions via the sitting-drop vapor-diffusion method in initial crystallization screening trials. Ultimately, the optimal conditions were found to be 22.5% (*w*/*v*) polyethylene glycol 4000, 0.125 M ammonium acetate, and 0.1 M sodium acetate trihydrate (pH 4.6) at 288 K. Initial attempts to determine the structure of *Ba* YknZ ED using a molecular replacement method with the program *MOLREP* [21] and structure of the YknZ homologue MacB (PDB code 3FTJ) [12] as a search model were unsuccessful. Fortunately, the structure of *Ba* YknZ ED was determined using SAD phasing methods with SeMet-substituted protein crystals. The best crystals readily diffracted to better than 2.0 Å resolution. From the diffraction dataset, the crystals were found to belong to the orthorhombic space group *P*2_1_2_1_2_1_, with unit-cell parameters of *a* = 44.2, *b* = 46.2, and *c* = 87.5 Å. Based on the estimated Matthews coefficient (1.8 Å^3^ Da^-1^) and the solvent-content prediction (31.66%), a monomer was expected in the asymmetric unit. The diffraction data set had a resolution range of 41.0–2.0 Å with 99.8% completeness, an *R*_merge_ of 28.8%, an R_factor_ of 19.44%, and an R_free_ of 24.73%. In the Ramachandran plot, 96.69% of the model residues were found in favored regions, 3.31% in allowed regions, and 0.00% in disallowed regions. The data collection and the refinement statistics for the structure are presented in Table 1.

### Overall structure of *Ba* YknZ ED

The refined model of *Ba* YknZ ED contains 188 residues from Asn^58^ to Asn^256^, and the N- and C-terminal regions of *Ba* YknZ ED were disordered in the crystal. The disordered N- and C-terminal regions of *Ba* YknZ ED may play a role in the connection between *Ba* YknZ ED and the transmembrane segments in full-length YknZ. The crystal asymmetric unit of the crystal contains one molecule of *Ba* YknZ ED. We also performed size-exclusion chromatography to gain insight into the *Ba* YknZ ED oligomeric state and found *Ba* YknZ ED was eluted as a monomer similar to *Actinobacillus actinomycetemcomitans* MacB periplasmic core domain (*Aa* MacB PCD), which is a non-canonical ABC-type transporter within Gram-negative bacteria [12], To understand whether *Ba* YknZ ED have an affinity toward each other at high concentrations, we tested the potential of *Ba* YknZ ED to dimerize in solution by native page electrophoresis, and analyses of native gels indicate that *Ba* YknZ ED can dimerize (S2 Fig). This result suggests that *Ba* YknZ ED molecules have an affinity toward each other, like other homologous proteins.

**Fig 2:**
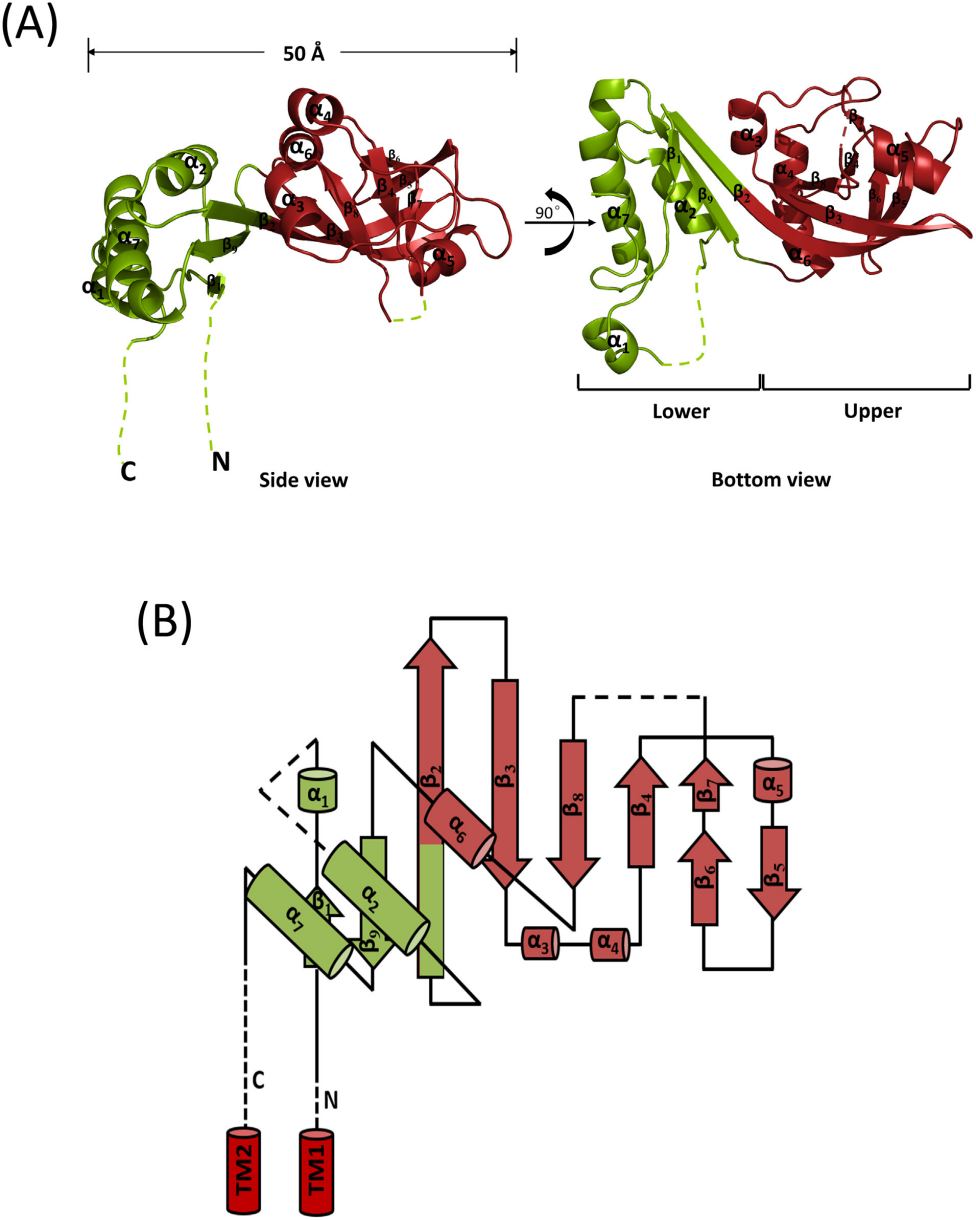
The structure of the *Ba* YknZ ED. (A) A *Ba* YknZ ED monomer in the asymmetric unit of the orthorhombic crystal form displayed as a cartoon representation. A side view and a bottom view are depicted. The disordered regions are shown with dashed lines. The upper and lower subdomains of the mixed α/β domain are shown in salmon and green, respectively. The secondary structural elements are numbered. (B) Topology diagram of *Ba* YknZ ED depicted in the same color scheme as in (A). Labeled loop regions are discussed in the text. Hypothetical transmembrane segments 1 and 2 (TM1 and TM2, respectively) are indicated with red cylinders.

The YknZ ED structure included a mixed α/β domain that was dispersed into upper and lower subdomains (Fig 2A). The upper subdomain was formed by residues 103–219 and was composed of a seven-stranded antiparallel β-sheet with four α-helices. A loop, in which significant electron density was not observed, connected β_2_ to β_9_ in this domain. The lower subdomain was formed by residues 58–102 and 219–256 and consisted of three α-helices and a three-stranded antiparallel β-sheet. A loop connected α_1_ to α_2_ in this subdomain and was disordered in the crystal structure of *Ba* YknZ ED. Additionally, no interactions were observed between the two distinct components, which were connected via a linker (β_2_ and β_9_) (Fig 2B).

### *Ba* YknZ ED molecular architecture is analogous to homologous protein structures

**Fig 3:**
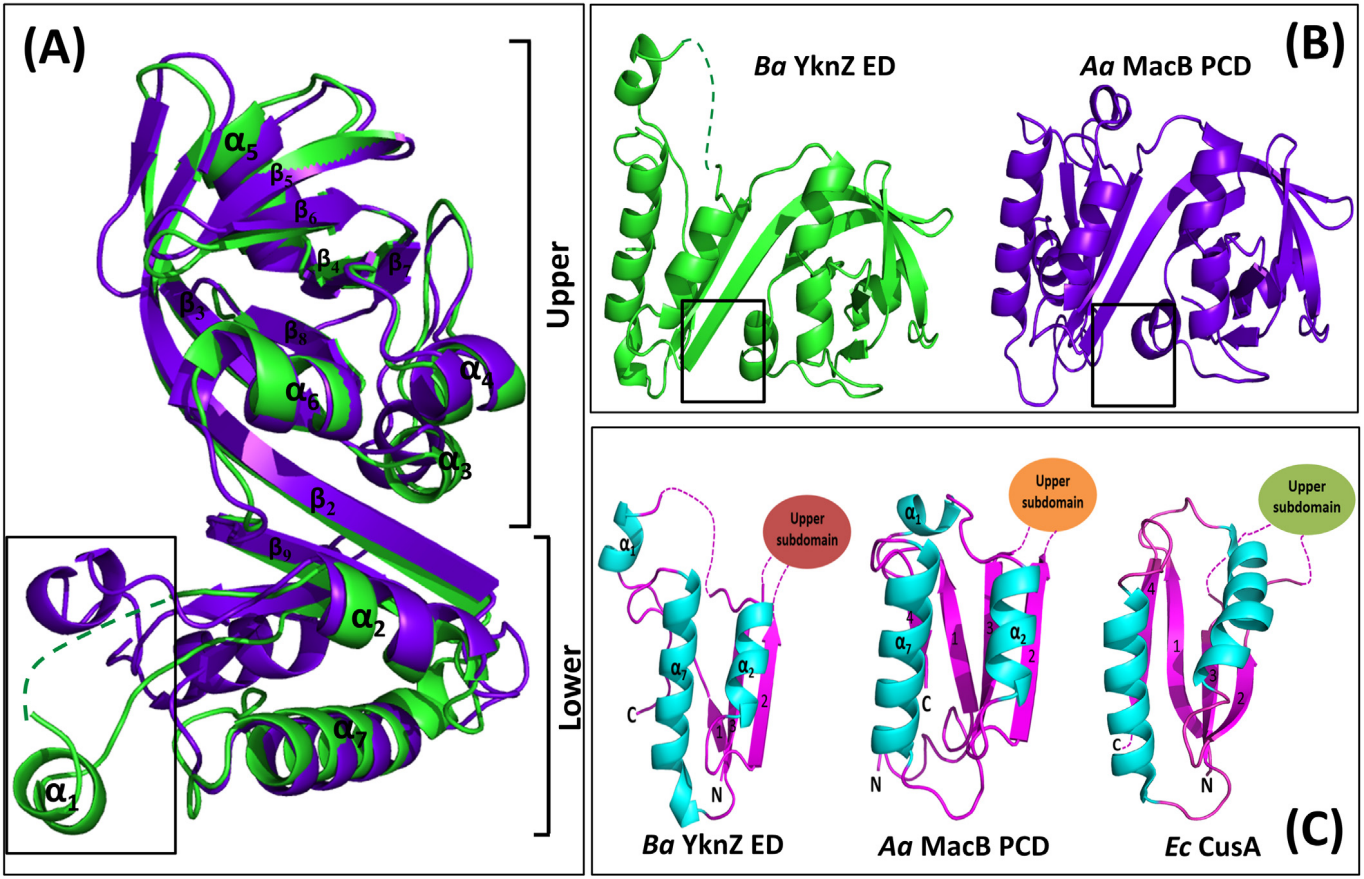
Structural comparison of *Ba* YknZ ED with other homologues. (A) Structural superposition of *Ba* YknZ ED and *Aa* MacB PCD. All residues were used for the superposition. The structures of *Ba* YknZ ED and *Aa* MacB PCD are shown in green and purple blue, respectively. The black box indicates the structurally different region between *Ba* YknZ ED and *Aa* MacB PCD. (B) Top views of *Ba* YknZ ED and *Aa* MacB PCD. The black box indicates the crevice between the upper and lower subdomains. (C) Lower subdomain of *Ba* YknZ ED, lower subdomain of *Aa* MacB PCD, and subdomains of the CusA pore domain (Bottom right, PDB code 3K07).

The DaliLite server [22] was used to identify unique proteins with structural similarity to *Ba* YknZ ED. The DaliLite server revealed that the structure of *Aa* MacB PCD was highly similar to *Ba* YknZ ED, which was found to be the top match and showed a Z-score of 20.1 and sequence identity of up to 22%. As evident from the results, the structural overlays of the two proteins revealed highly similar overall topologies with a root mean square deviation (rmsd) of 1.6 Å between the corresponding 121 residues. Upon superposition of two proteins as references, we found the overall structures and relative domain orientations of Ba YknZ ED and Aa MacB PCD to be very similar but not identical; some minor structural differences were present (Fig 3, Left). In particular, the structure of *Aa* MacB PCD revealed that each monomer had a sickle shape that was dispersed into the following two distinct components: (1) the N- and C-terminal regions and (2) a mixed α/β domain [12]. In contrast, the structure of *Ba* YknZ ED only contained a mixed α/β domain with disordered N- and C-terminal regions. Additionally, the *Aa* MacB PCD β_6_ was divided into two β-strands (β_6_ and β_7_) in the *Ba* YknZ ED structure. We previously found a shallow crevice in the MacB PCD structure; the mixed α/β domain is almost completely divided into two parts by this crevice. We also found this crevice in the middle of the mixed α/β domain in the *Ba* YknZ ED structure, where it virtually divided the domain into two parts (Fig 3, Upper-right). The overall structural architecture of the lower subdomain of *Aa* MacB PCD was similar to that of subdomains in the AcrB porter domain [12].

To understand whether the overall structural architecture of the *Ba* YknZ ED lower subdomain had high similarity to other homologous proteins, we compared the structure of the *Ba* YknZ ED lower subdomain with the *Aa* MacB PCD lower subdomain and the subdomains of the inner membrane transporter CusA pore domain from *E*. *coli* [23]. We found that the domain structures were generally similar, although there were slight differences. In Gram-negative bacteria, CusA is the inner membrane transporter of the heavy metal efflux pump CusCBA [23]. The most significant structural difference is the number of β-strands; the lower subdomain of *Ba* YknZ ED contained three β-strands, while the other structures each contain four β-strands. Whereas α_1_ and α_2_ were connected in the *Aa* MacB PCD structure, the corresponding region in the *Ba* YknZ ED structure was largely disordered, and the conformations of these helices were slightly different in *Ba* YknZ ED, as α_1_ was tilted outwards compared with the *Aa* MacB PCD structure. We also observed that β_1_ in the *Ba* YknZ ED N-terminal region was shorter than that of the other homologous proteins (Fig 3, lower-right).

### *Ba* YknZ ED can directly bind to YknX

**Fig 4:**
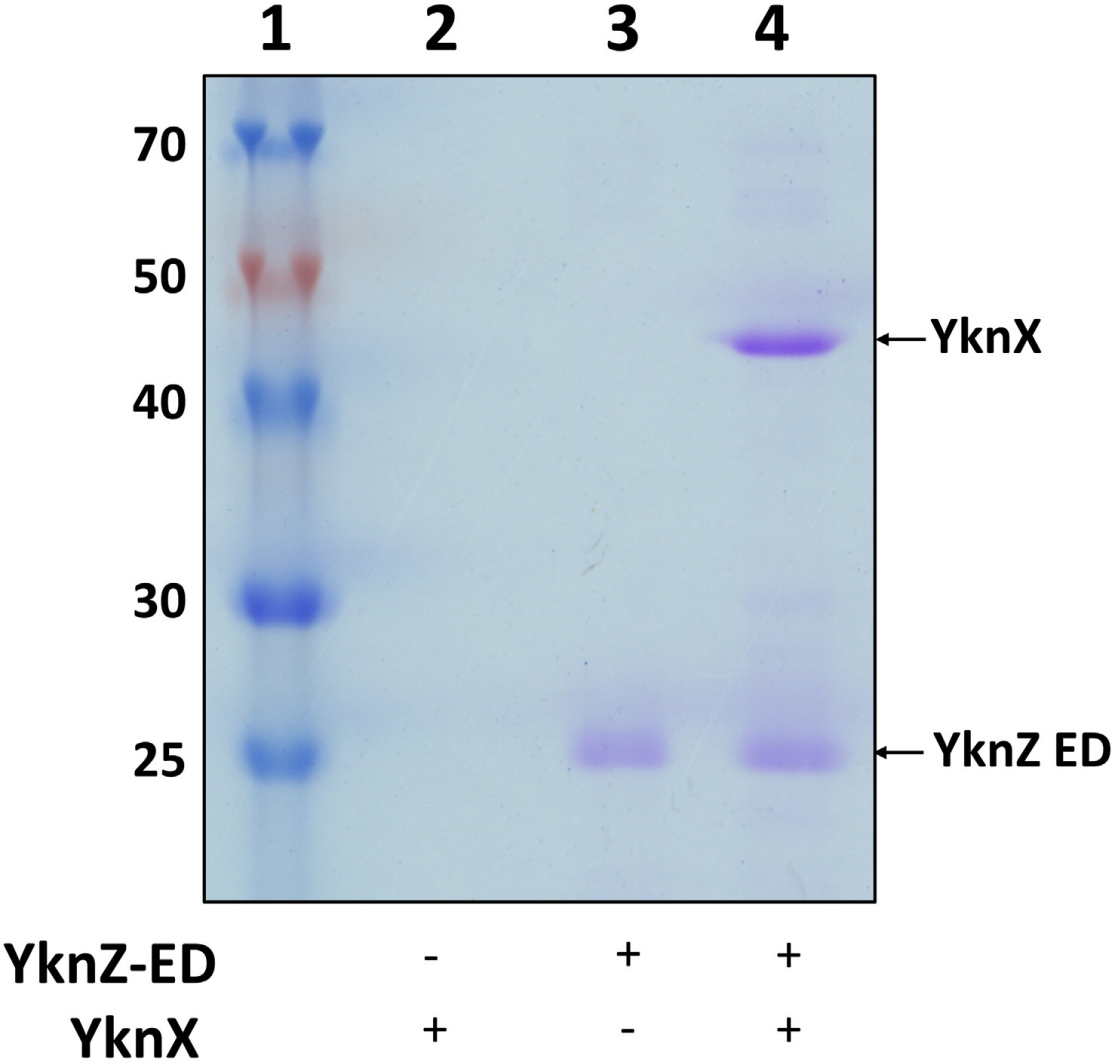
Direct interaction between YknZ ED and YknX. *In vitro* binding assay between *Ba* YknZ ED and *Ba* YknX. *Ba* YknZ ED-coupled CNbr-activated resin and *Ba* YknZ ED-uncoupled CNbr-activated resin were preincubated with purified YknX protein and washed to remove unbound proteins. Finally, the bound proteins were analyzed on an SDS-polyacrylamide gel. Lane 1, molecular weight markers (labeled in kDa); lane 2, after the preincubation of *Ba* YknZ ED-uncoupled CNbr-activated resin with the YknX protein; lane 3, non-incubated *Ba* YknZ ED-coupled CNbr-activated resin; lane 4, after the preincubation of *Ba* YknZ ED-coupled CNbr-activated resin with YknX.

Yamada et al. used chemical cross-linking and co-purification experiments to demonstrate that full-length YknYZ can complex with YknX [4]. To test whether *Ba* YknZ ED directly binds YknX, a binding assay was performed using purified YknZ ED and YknX proteins, as described in the Materials and Methods section. As shown in Fig 4, the purified YknX protein was not bound to the CNbr-activated region, which is in sharp contrast to the strong binding of YknX to YknZ ED. This result indicated that *Ba* YknZ ED directly associated with YknX, which was consistent with a previous report in which full-length YknYZ was used [4].

## Discussion

In both Gram-positive and Gram-negative bacteria, ABC-type transporters are functional subunits of multi-component transporters that perform diverse physiological functions utilizing ATP hydrolysis as a driving force. Recently, an ABC transporter YknYZ and its homologues were identified in various bacteria [4,10]. Unlike most dimeric ABC-type transporters, MacB and YknYZ are characterized as non-canonical ABC-type transporters, which consist of a four-transmembrane segment, an N-terminal nucleotide binding domain (NBD), and a large periplasmic domain [7,24]. In this study, we presented the first three-dimensional structure of the *Ba* YknZ extracellular domain, which is associated with YknY. The overall structure of the *Ba* YknZ ED monomer showed a structure similar to that of *Aa* MacB PCD. The sickle shape YknZ ED structure included a mixed α/β domain that was dispersed into upper and lower subdomains by a shallow crevice, and the structure of the *Ba* YknZ ED lower subdomain was generally similar to those in other homolog proteins such as the *Aa* MacB PCD lower subdomain and the subdomains of the inner membrane transporter CusA pore domain from *E*. *coli*. The upper subdomain of MacB or CusA may play a role in forming similar structures in the cognate binding partners of MacB or CusA [12]; thus, we speculate that the upper subdomain of YknZ fits into YknX and the lower subdomain of YknZ provides the substrate entrance site.

**Fig 5:**
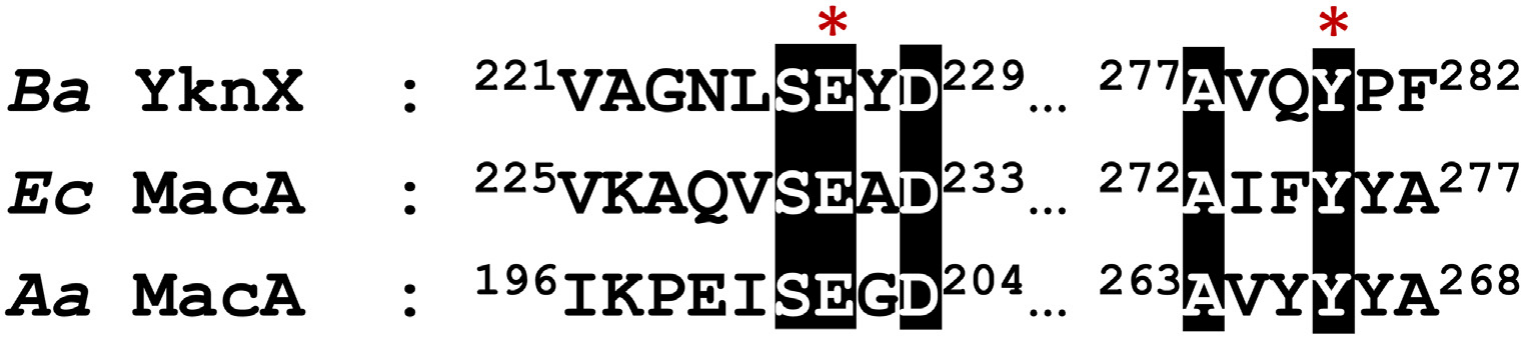
The sequence alignment of MFPs from different species. The *Ba* YknX Gle^227^ and Tyr^280^ are strictly conserved; the conserved residues are highlighted in black, as shown in the sequence alignment of MFPs from different species: *B*. *amyloliquefaciens* (*Ba*), *E*. *coli* (*Ec*), *A*. *actinomycetemcomitans* (*Aa*).

**Fig 6:**
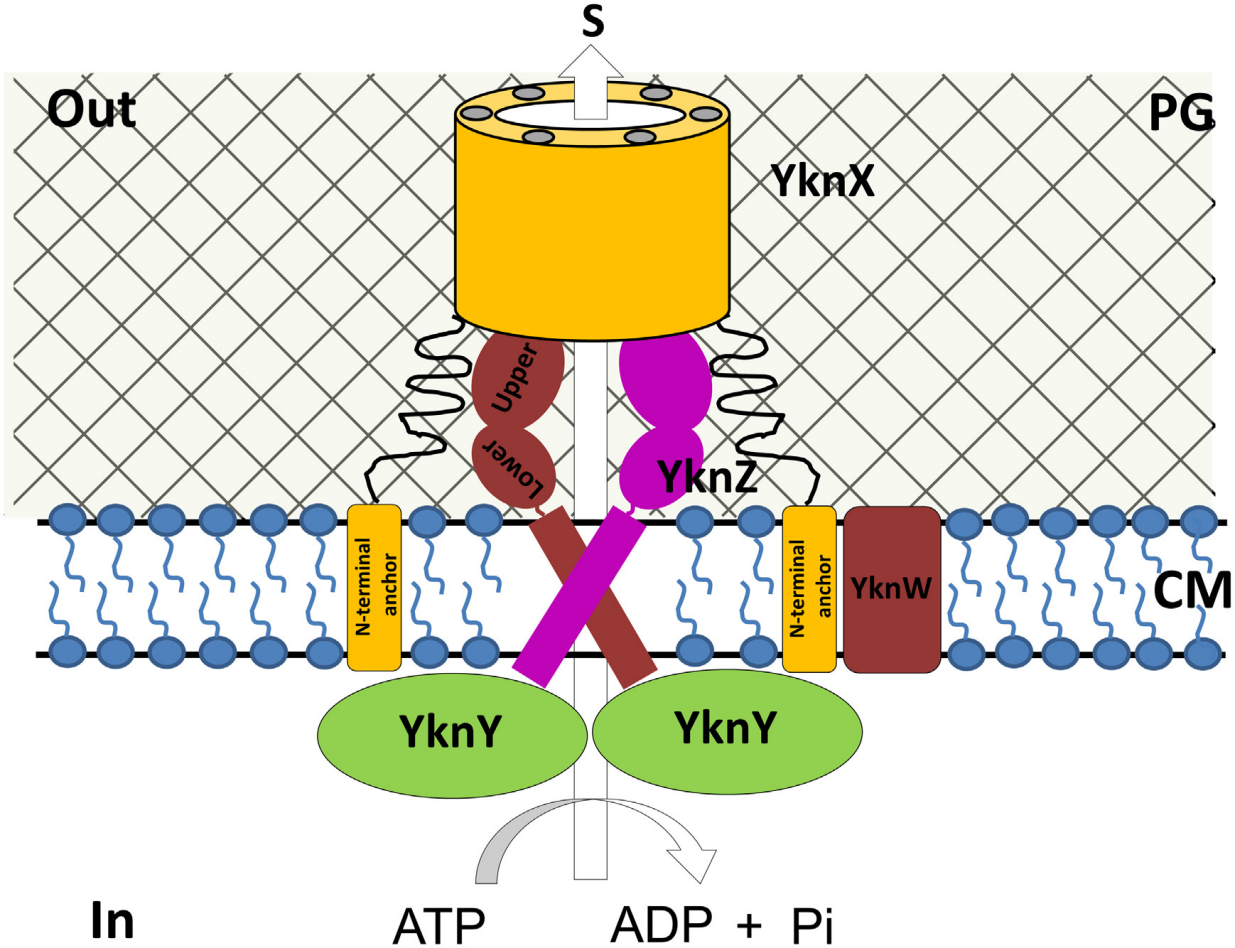
Schematic representation of the YknWXYZ four-component transporter. *Ba* YknX resembles a funnel, and the N-terminal transmembrane segments are shown as rectangles in the cell membrane. The cell membrane portion of YknZ is accommodated by the hollow adjacent to the membrane, and the YknZ ED is directly bound to *Ba* YknX. YknY contributes to dimerization, which may also contribute to *Ba* YknZ dimerization. *Ba* YknW contributes to dimerization and modulates complex assembly. The four-component transporter YknWXYZ transports substrates by utilizing ATP hydrolysis as a driving force across the cytoplasmic membrane to the extracellular space. The complex spans the intracellular space (In), the cytoplasmic membrane (CM), the extracellular space (Out), and the substrate (S).

Recently, assembly models for the MacAB-TolC and CusCBA pumps have been proposed [13,22]. In these models, the inner membrane component (MacB and CusA, respectively) directly interacts with MFP (MacA and CusB, respectively), which acts as a hexameric protein. However, these previous studies mentioned above did not reveal direct interactions between the YknZ dimer and the YknX oligomer or indicate which part of YknZ is important for YknX binding. In this study, we observed an interaction between YknZ ED and YknX using an in vitro binding assay, which was consistent with a previous report in which full-length YknYZ was used [4]. The MFP YknX is crucial for the functional assembly of the YknWXYZ transporter in the extracellular. Moreover, the structure and overall size of the YknX protein are similar to those of MacA (sequence identity 32%) (S3 Fig). In our previous work, we suggested that MacA is a funnel-like hexameric protein [9]. In this study, we observed that the *Ba* YknX residues Gle^227^ and Tyr^280^ were strictly conserved in the sequence alignment (Fig 5); these two residues are located within the β-barrel domains and play an important role in hexamerization in the crystal structure of MacA [9]. We speculate that YknX forms a similar oligomeric assembly in the functional complex despite previous biochemical data indicating that YknX likely forms a dimer or trimer [4]. In this study, we also found that YknZ ED can directly interact with MFP YknX, but we did not determine which region of YknZ is important for this interaction. Further structural and functional studies of the binary and ternary complexes (YknXYZ) are required to determine whether the upper subdomains of YknZ are essential for the interactions between YknZ and YknX and whether these interactions are similar or different in the assembly of the four-component transporter YknWXYZ. In addition, a previous study found that the individual homodimeric protein YknY can associate with YknZ, and the assembly of the complex is modulated by YknW in the YknWXYZ complex [4]. Based on these findings, we propose an assembly model in which the YknZ ED dimer directly fits into the hexameric YknX, which displays an upside-down, funnel-shaped orientation (Fig 6). Given the results presented in this study, we cannot draw conclusions regarding the relationship between the crystal structure of *Ba* YknZ ED and SDP. However, we speculate that the upper subdomain of YknZ fits into the funnel-mouth region of the YknX hexamer and that the lower subdomain of YknZ provides the SDP entrance site.

In conclusion, we purified YknZ ED and successfully determined the crystal structure of *Ba* YknZ ED at a resolution of 2.0 Å. Our structure of YknZ showed that each monomer includes a mixed α/β domain, which is dispersed into upper and lower subdomains in a manner similar to that observed with *Aa* MacB PCD. We also found that the *Ba* YknZ ED crystal structure was very similar to that of *Aa* MacB PCD. In vitro binding assays demonstrated that YknZ ED had an affinity toward MFP YknX. The YknX hexamer displayed a upside-down, funnel-shaped orientation similar to *Aa* MacA. Based on the above results, we proposed an assembly model for YknWXYZ that can offer a structural basis for understanding cannibalistic peptide toxin resistance in *B*. *amyloliquefaciens*. However, crystal structures of the complex are needed to reveal the assembly of this type of transporter at the atomic level.

## Supporting Information

S1 Fig Overexpression and purification of *Ba* YknZ ED. *Lane* 1, Molecular-weight markers (labeled in kDa); *lane* 2, uninduced cell supernatants; *lane* 3, 0.5 mM IPTG-induced cell supernatants; *lane* 4, the pooled fractions eluted from a Nickel-ion affinity column; *lane* 5, after the treatment with the TEV protease; lane 6, the final sample.
(DOCX)

S2 Fig Native page electrophoresis of purified *Ba* YknZ ED. *Ba* YknZ ED protein was subjected to 15% native polyacrylamide gels and stained with Coomassie blue. *Lane* 1, Molecular-weight markers (labeled in kDa); *lane* 2, 5μg *Ba* YknZ ED sample.
(DOCX)

S3 Fig Sequence alignment of the *Ba* YknX, and *Aa* MacA sample. The alignment was performed using CLUSTALX^20^. Strictly conserved residues are highlighted in black.
(DOCX)
