# Supplementary material for: The Crystal Structure of the YknZ Extracellular Domain of ABC Transporter YknWXYZ from Bacillus amyloliquefaciens
Source: PLoS One. 2016 May 31;11(5):e0155846. doi: 10.1371/journal.pone.0155846 (PMC4887032; doi:10.1371/journal.pone.0155846)

**S1 Fig. Overexpression and purification of *Ba* YknZ ED.**

*Lane* 1, Molecular-weight markers (labeled in kDa); *lane* 2, uninduced cell supernatants; *lane* 3, 0.5 mM IPTG-induced cell supernatants; *lane* 4, the pooled fractions eluted from a Nickel-ion affinity column; *lane* 5, after the treatment with the TEV protease; lane 6, the final sample.


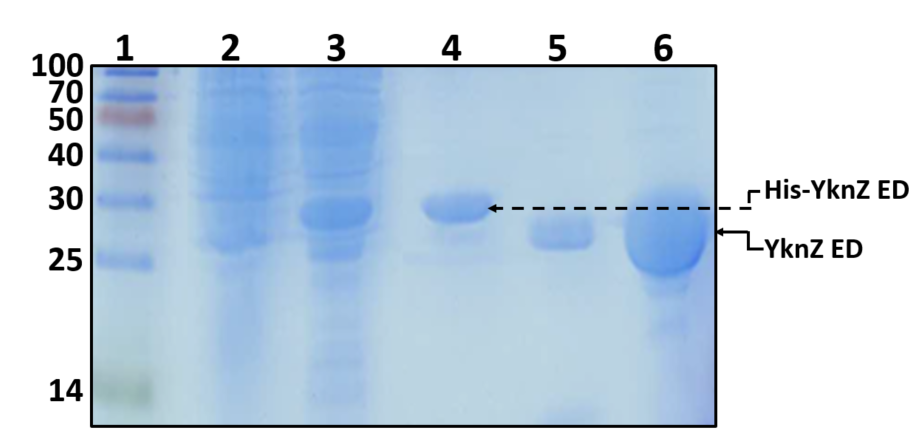

Supplement: S1 Fig — Lane 1, Molecular-weight markers (labeled in kDa); lane 2, uninduced cell supernatants; lane 3, 0.5 mM IPTG-induced cell supernatants; lane 4, the pooled fractions eluted from a Nickel-ion affinity column; lane 5, after the treatment with the TEV protease; lane 6, the final sample. (DOCX) [file pone.0155846.s001.docx]
