# Supplementary material for: The Crystal Structure of the YknZ Extracellular Domain of ABC Transporter YknWXYZ from Bacillus amyloliquefaciens
Source: PLoS One. 2016 May 31;11(5):e0155846. doi: 10.1371/journal.pone.0155846 (PMC4887032; doi:10.1371/journal.pone.0155846)

**S2 Fig. Native page electrophoresis of purified *Ba* YknZ ED.**

*Ba* YknZ ED protein was subjected to 15% native polyacrylamide gels and stained with Coomassie blue. *Lane* 1, Molecular-weight markers (labeled in kDa); *lane* 2, 5μg *Ba* YknZ ED sample.


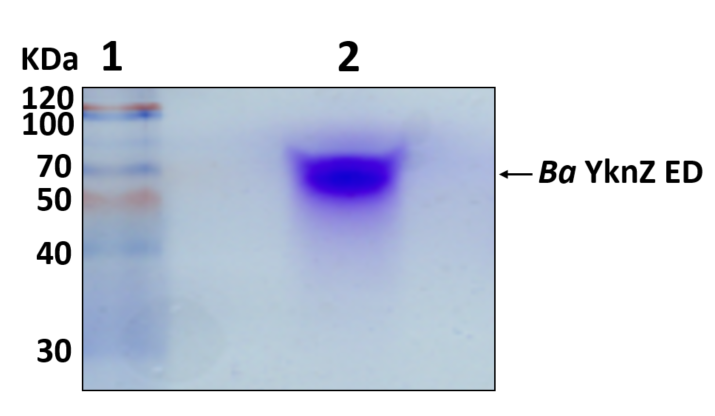

Supplement: S2 Fig — Ba YknZ ED protein was subjected to 15% native polyacrylamide gels and stained with Coomassie blue. Lane 1, Molecular-weight markers (labeled in kDa); lane 2, 5μg Ba YknZ ED sample. (DOCX) [file pone.0155846.s002.docx]
