# Supplementary material for: The Crystal Structure of the YknZ Extracellular Domain of ABC Transporter YknWXYZ from Bacillus amyloliquefaciens
Source: PLoS One. 2016 May 31;11(5):e0155846. doi: 10.1371/journal.pone.0155846 (PMC4887032; doi:10.1371/journal.pone.0155846)
Supplement: S3 Fig — The alignment was performed using CLUSTALX20. Strictly conserved residues are highlighted in black. (DOCX) [file pone.0155846.s003.docx]

**S3 Fig. Sequence alignment of the *Ba* YknX, and *Aa* MacA sample.**

The alignment was performed using CLUSTALX[^20^](#_ENREF_20). Strictly conserved residues are highlighted in black.

**
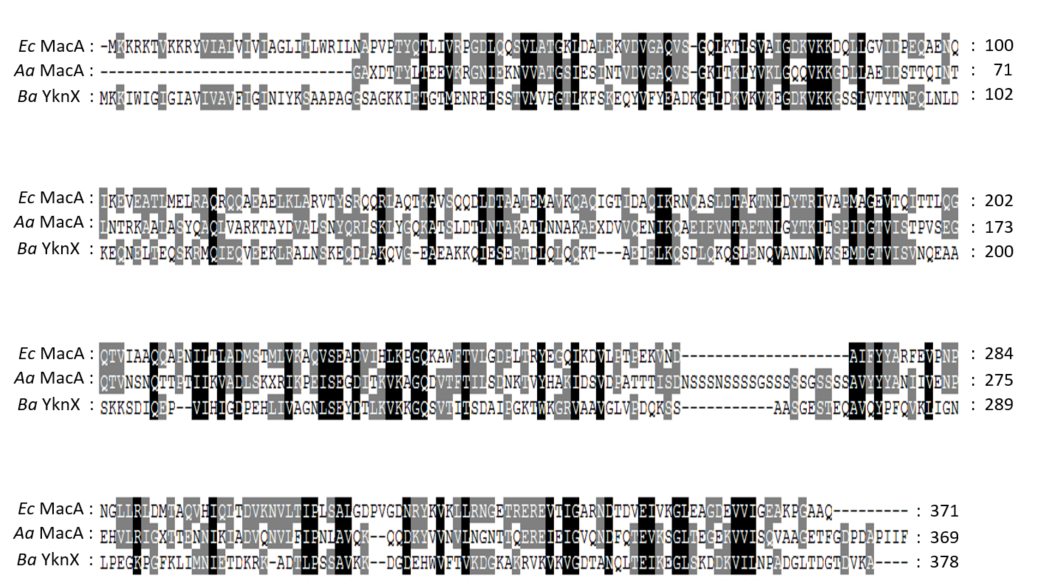
**
